# Neutrophils do not impact viral load or the peak of disease severity during RSV infection

**DOI:** 10.1038/s41598-020-57969-w

**Published:** 2020-01-24

**Authors:** Freja Kirsebom, Christina Michalaki, Marina Agueda-Oyarzabal, Cecilia Johansson

**Affiliations:** National Heart and Lung Institute, Imperial College London, London, UK

**Keywords:** Viral infection, Acute inflammation, Mucosal immunology

## Abstract

Lung and airway neutrophils are a hallmark of severe disease in infants with respiratory syncytial virus (RSV)-induced lower respiratory tract infections. Despite their abundance in the lungs during RSV infection of both mice and man, the role of neutrophils in viral control and in immune pathology is not clear. Here, antibody mediated neutrophil depletion was used to investigate the degree to which neutrophils impact the lung immune environment, the control of viral replication and the peak severity of disease after RSV infection of mice. Neutrophil depletion did not substantially affect the levels of inflammatory mediators such as type I interferons, IL-6, TNF-α or IL-1β in response to RSV. In addition, the lack of neutrophils did not change the viral load during RSV infection. Neither neutrophil depletion nor the enhancement of lung neutrophils by administration of the chemoattractant CXCL1 during RSV infection affected disease severity as measured by weight loss. Therefore, in this model of RSV infection, lung neutrophils do not offer obvious benefits to the host in terms of increasing anti-viral inflammatory responses or restricting viral replication and neutrophils do not contribute to disease severity.

## Introduction

Respiratory syncytial virus (RSV) is a major global pathogen that causes annual epidemics worldwide^1,2^. RSV typically infects the upper respiratory tract causing mild disease in healthy adults. However, certain vulnerable populations, such as immunocompromised individuals, the elderly and infants, are susceptible to RSV-induced lower respiratory tract infections (LRTI) causing bronchiolitis and pneumonia. Severe RSV disease occurs most commonly in infants and RSV infection is the single greatest cause of infant hospitalizations in the developed world^3,4^. Currently, there are no effective drugs or vaccines against RSV and treatment consists of mechanical ventilation in intensive care units^5^. RSV infection has major consequences for respiratory health in both early and later life as infants who overcome RSV induced LRTIs are more susceptible to wheeze^6,7^ and asthma^8,9^. There is therefore a need for a better understanding of the factors that drive severe RSV disease.

It is well established that the host immune response to RSV is a major determinant of disease severity^10^. The presence of increased number of neutrophils in the lung is a hallmark of severe RSV disease in both mouse and man^11,12^ and neutrophils can constitute >90% of the cellular compartment of the bronchoalveolar lavage (BAL) during RSV-induced bronchiolitis in infants^11^. Neutrophils are phagocytic cells that can be activated to degranulate and secrete a multitude of pre-stored proteolytic enzymes and anti-microbial peptides, undergo oxidative burst and even secrete their own chromatin in a unique form of cell death to form pathogen-trapping, web-like structures termed neutrophil extracellular traps (NETs)^13–15^. The recruitment and activation of neutrophils must be tightly regulated to minimize bystander damage, a cause of immunopathology during both infectious disease and sterile inflammation^16,17^. Neutrophils are particularly important in the defense against bacterial and fungal infections^13^ but their contribution to host defense during viral infections is less clear as the intracellular nature of viruses inherently make them less vulnerable to extracellular methods of attack. However, circulating neutrophils are a predictor of pneumonia risk in patients with chronic obstructive pulmonary disease^18^ and, in a mouse model of allergic airway hypersensitivity, rhinovirus can trigger NETosis, inducing type 2 airway responses and lung pathology^19^. Furthermore, antibody mediated neutrophil depletion has been used to demonstrate that neutrophils can be both beneficial and detrimental during influenza virus and pneumonia virus of mice (PVM) infection suggesting that the role of neutrophils is complex and may display mouse and virus strain-specific differences^20–23^.

Studies of infants with severe RSV disease have implicated neutrophils in disease pathogenesis^24,25^. In a transcriptomic analysis of blood from children with RSV-induced bronchiolitis, transcripts related to neutrophil function were in the top ten over-represented genes^26^. Furthermore, airway IL-8, a neutrophil chemoattractant, correlates with the duration of hospital stay of infants with bronchiolitis^27^ and the concentration of neutrophil elastase (NE), an enzyme stored in azurophilic granules and secreted during activation, is elevated in both the serum and nasal lavage of children with confirmed RSV^24^. Finally, a genetic polymorphism in the region upstream of the IL-8 gene, associated with increased IL-8 production, has been found to be more prevalent in infants hospitalized with RSV-induced bronchiolitis compared to controls^28^.

To study the contribution of neutrophils to anti-viral immunity and pathology, we manipulated the cells *in vivo* during acute RSV infection. Neutrophil depletion did not affect the overall early inflammatory landscape in the lungs, viral replication or the timing or severity of disease. Similarly, increasing the number of neutrophils by administration of the neutrophil chemoattractant CXCL1 either early during the infection or later at the peak of disease did not contribute to disease severity as measured by weight loss. Our findings support recent literature^29–31^ that in many inflammatory contexts the potentially pathogenic bystander effects of neutrophils can be controlled and tolerated, even by a structurally delicate tissue such as the lung.

## Results

### Neutrophil depletion does not have a major impact the pro-inflammatory environment in the lung early during RSV infection

**Figure 1:**
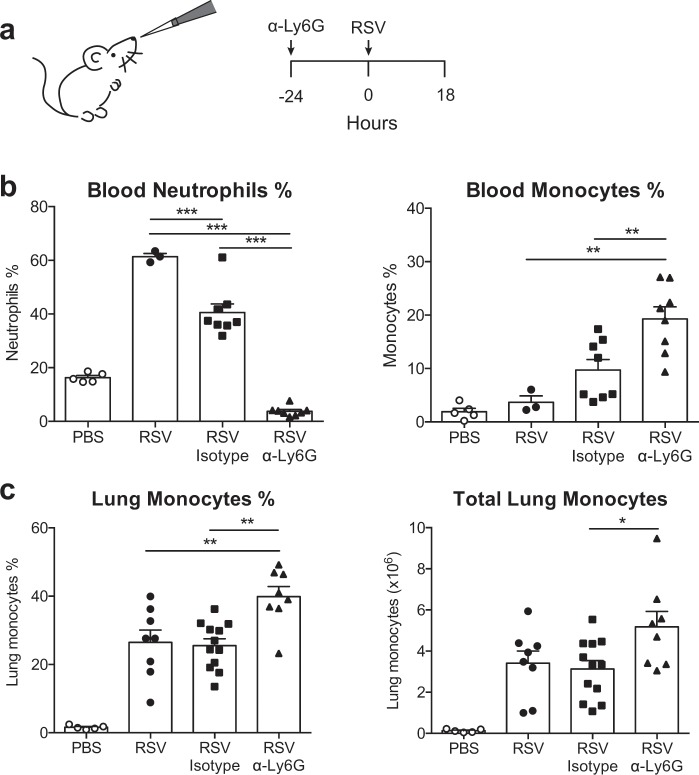
Antibody mediated (α-Ly6G) neutrophil depletion does not impair monocyte recruitment 18 h post RSV infection. (**a**) Wt mice were mock (PBS) or RSV infected for 18 h. Mice were given 200 µg i.n. and 500 µg i.p. α-Ly6G or isotype control antibody day −1. (**b**) Frequencies of neutrophils and monocytes in the blood. (**c**) Frequency and total number of monocytes in the lung (Supp. Figure 1 for gating strategy). Data are presented as the mean ± SEM from 5 (PBS), 3 (RSV) or 8–12 (RSV+ antibody treatment) individual mice pooled from two or three independent experiments. Each symbol represents an individual mouse. Statistical significance of differences was determined by one-way ANOVA with Tukey’s post hoc test. Only the statistical significances between RSV infected groups are shown. *P ≤ 0.05, **P ≤ 0.01, ***P ≤ 0.001.

To investigate the role of neutrophils during RSV infection, antibody-mediated (α-Ly6G) neutrophil depletion was used (Fig. 1a). At 24 h pre-infection, mice were either treated with isotype control antibody or with neutrophil depleting (α-Ly6G) antibody (Fig. 1a), an established strategy that depletes neutrophils for two days^20,23,32^. Mice were intranasally (i.n.) infected with RSV and neutrophil depletion confirmed in airways (BAL^33^;) and blood (Fig. 1b) at 18 h post-infection (p.i.; see Supp. Figure 1 for gating strategy), the peak of neutrophil infiltration^12,33^. RSV infected, isotype control antibody treated mice had a lower frequency of circulating neutrophils in the blood compared to RSV only mice (Fig. 1b) but were able to recruit neutrophils to the airways^33^.

**Figure 2:**
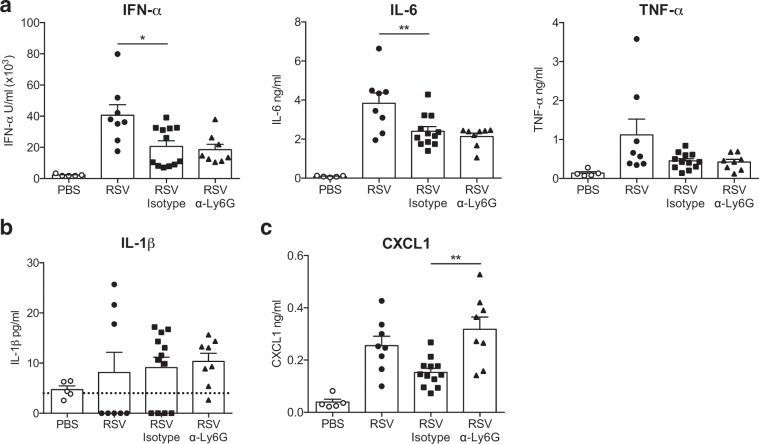
Neutrophils do not contribute to the induction of a pro-inflammatory immune environment in the lungs 18 h post RSV infection. Wt mice were mock (PBS) or RSV infected for 18 h. To deplete neutrophils, RSV infected mice were given 200 µg i.n. and 500 µg i.p. α-Ly6G or isotype control antibody day −1. (**a**) IFN-α, IL-6 and TNF-α. (**b**) IL-1β and (**c**) CXCL1 were quantified in the BAL by ELISA. Data are presented as the mean ± SEM from 5 (PBS) or 8–12 (RSV) individual mice pooled from two or three independent experiments. Each symbol represents an individual mouse. The dotted line in (**b**) represents the detection limit. Statistical significance of differences was determined by one-way ANOVA with Tukey’s post hoc test. Only the statistical significances between RSV infected groups are shown. *P ≤ 0.05, **P ≤ 0.01, ***P ≤ 0.001.

In addition to neutrophils, anti-viral monocytes are recruited to the lung early during RSV infection^12^. Interestingly, both the frequency of blood monocytes (Fig. 1b) and the frequency and total number of recruited Ly6C^+^ CD64^+^ inflammatory monocytes were increased in α-Ly6G treated mice compared to isotype control treated mice (Fig. 1c). Next, we assessed how neutrophil depletion affects the production of pro-inflammatory immune mediators in the airways in response to RSV (Fig. 2). Neutrophil depletion did not affect the levels of the pro-inflammatory mediators IFN-α, IL-6, TNF-α or IL-1β in the airways at 18 h p.i. when compared to RSV infected, isotype control treated mice (Fig. 2a,b). Isotype control treated, RSV infected mice did accumulate less IFN-α and IL-6 in the airways compared to untreated, infected mice suggesting that the isotype control antibody treatment may have some unspecific effects. Interestingly, concentrations of CXCL1 (and *Cxcl1* mRNA), a major neutrophil chemoattractant^34^, were significantly increased in the airways of RSV infected, α-Ly6G treated mice (Fig. 2c and Supp. Figure 2a). We also assessed the expression of transcripts encoding inflammatory mediators such as *Cxcl2, Cxcl12, Il1a* and *Il1b*, which again showed that it was unaffected by neutrophil depletion (Supp. Figure 2a,b). Finally, neutrophil depletion during RSV infection did not affect the induction of *Il33, Areg* and *Ptgs2*, representing alarmins and mediators associated with tissue repair (Supp. Figure 2c), and did not impact expression of genes associated with mucus production (*Muc5ac*; Supp. Figure 2d).

### Neutrophils do not restrict RSV replication *in vivo*

**Figure 3:**
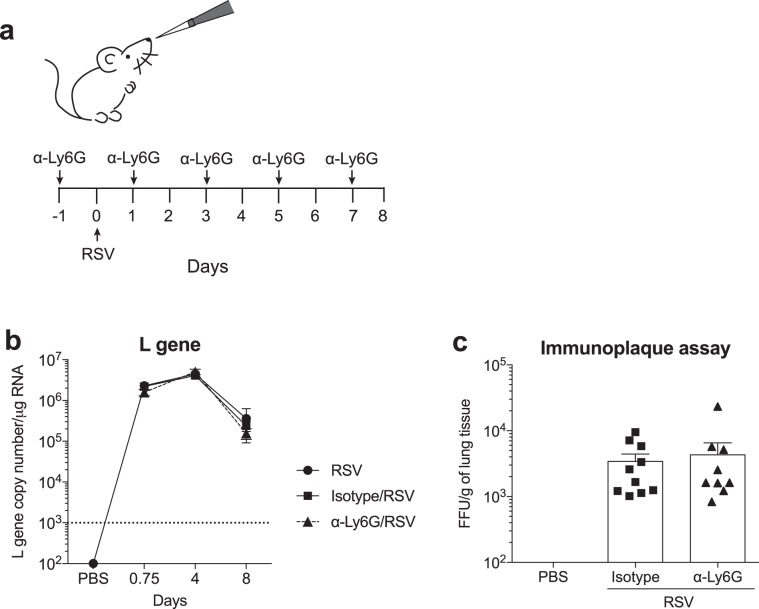
Neutrophils do not restrict viral replication during RSV infection. (**a**) C57BL/6 or wt mice were mock (PBS) or RSV infected for 18 h, 4 and 8 days. To deplete neutrophils, 150 µg i.p. α-Ly6G or isotype control antibody was administered on days −1, 1, 3, 5 and 7. (**b**) RSV L gene copy number was quantified in lung tissue by RT-qPCR. Copy numbers were determined using a plasmid standard and the results were normalized to *Gapdh* levels. (**c**) Infectious viral particles in the lung on day 4 were quantified by immunoplaque assay. Data from (**b)** are presented as the mean ± SEM from 25 PBS (pooled from each time point) and 8–12 RSV (18 h and day 4) pooled from two independent experiments or 4–5 individual mice from one (day 8) experiment. Statistical significance of differences was determined by two-way ANOVA with Bonferroni’s post hoc test. Data from (**c)** are presented as the mean ± SEM of 9 (PBS) or 10 (RSV isotype/α-Ly6G) individual mice pooled from two independent experiments. Each symbol represents an individual mouse. Statistical significance of differences was determined by one-way ANOVA with Tukey’s post hoc test.

Neutrophils prevent the replication and spread of many bacteria and fungi but much less is understood about their ability to restrict the replication of viruses^35^. To investigate if neutrophils contribute to host defense by restricting RSV replication in the lungs, viral load in the lungs 18 h, 4 and 8 days p.i. was assessed by quantifying RSV L gene by RT-qPCR (Fig. 3). For antibody mediated neutrophil depletion, mice were treated with α-Ly6G or isotype control one day pre-infection as well as every second day after the infection (Fig. 3a). For these experiments a lower dose of the α-Ly6G and isotype antibodies was used (Supp. Figure 3 and^22^). There was no difference in RSV L gene copy number between the RSV only group, the RSV-infected isotype treated mice or neutrophil depleted mice at any of the time points studied (Fig. 3b). As quantifying RSV L gene by RT-qPCR does not accurately quantify the number of replication-competent RSV particles, we also assessed viral load by immunoplaque assay at day 4 p.i. (Fig. 3c), the peak of viral load in the mouse model^36^. There was no significant difference in the number of replication-competent RSV particles quantified in the lungs of isotype control treated and α-Ly6G treated RSV infected mice (Fig. 3c), confirming that neutrophils do not contribute to viral control during RSV infection.

### Neutrophils infiltrating the lung and airway does not directly contribute to disease severity in mice infected with RSV

**Figure 4:**
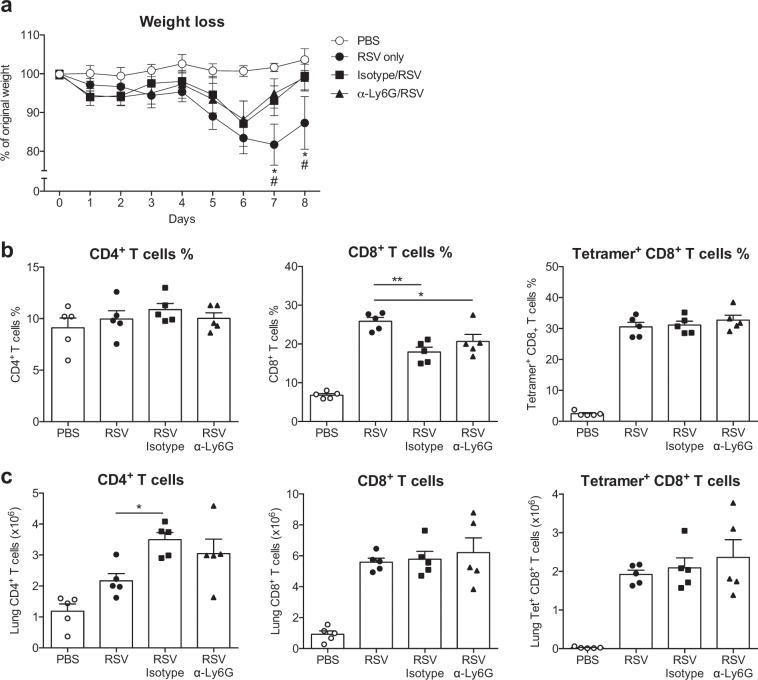
Neutrophils do not influence the recruitment of T cells to the lung on day 8 post RSV infection. C57BL/6 mice were mock (PBS) or RSV infected. To deplete neutrophils, 150 µg i.p. α-Ly6G or isotype control antibody was administered on day −1 and on every second day throughout the infection. (**a**) Weight loss shown as the percentage of original weight. Frequencies (**b**) and total numbers (**c**) of CD19^−^ CD3^+^ CD4^+^ and CD8^+^ T cells day 8 p.i. RSV-specific CD8^+^ T cells were detected by staining with M_187–195_ tetramer (Supp. Figure 1 for gating strategy). Each symbol represents an individual mouse. Data are presented as the mean ± SEM of 5 individual mice per group from one experiment. Statistical significance of differences was determined by one-way ANOVA with Tukey’s post hoc test. Only the statistical significances between RSV infected groups are shown. *P ≤ 0.05, **P ≤ 0.01.

As neutrophil recruitment did not appear to be beneficial to the host by limiting RSV replication, we next wanted to assess whether it was detrimental by driving or enhancing disease severity (Fig. 4). Neutrophil recruitment to the lung is very transient; recruitment peaks early at 18 h p.i. and neutrophils are largely absent by 36 h p.i^12,33^. Therefore, mice were treated with α-Ly6G one day pre-infection as well as one day p.i. to ensure adequate depletion up to 72 h p.i. (Supp. Figure 4a). To quantify disease severity, weight loss was measured throughout as this is a well-established readout of severity of RSV infection^37^. Neutrophil depletion early during RSV infection of C57BL/6 mice did not impact weight loss throughout the infection (Supp. Figure 4b). We confirmed this finding by repeating the experiment in BALB/c mice, a strain more susceptible to RSV than C57BL/6 mice^38–40^. This again showed no difference in weight loss between α-Ly6G or isotype antibody treatment (Supp. Figure 4b).

Although the peak of lung neutrophils occurs early during RSV infection, neutrophils are also recruited into the lung during disease on days 5–7 p.i. concurrently with T cells, which are the primary cause of weight loss^41^. To confirm that neither the early neutrophil influx nor the T cell associated neutrophil influx contribute to weight loss during RSV infection, neutrophils were depleted throughout the infection by treatment with α-Ly6G every other day, starting at day -1 (Fig. 3a). There was no difference in weight loss between α-Ly6G treated and isotype control treated mice during the infection even if untreated RSV infected mice lost more weight on days 7 and 8 p.i. than either isotype control or α-Ly6G treated RSV infected mice (Fig. 4a). Neither the frequency nor total number of CD4^+^ or CD8^+^ T cells was affected by neutrophil depletion during RSV infection (Fig. 4b,c). Furthermore, neutrophil depletion did not influence the frequency or total number of RSV-specific (tetramer^+^) CD8^+^ T cells on day 8 p.i. (Fig. 4b,c).

**Figure 5:**
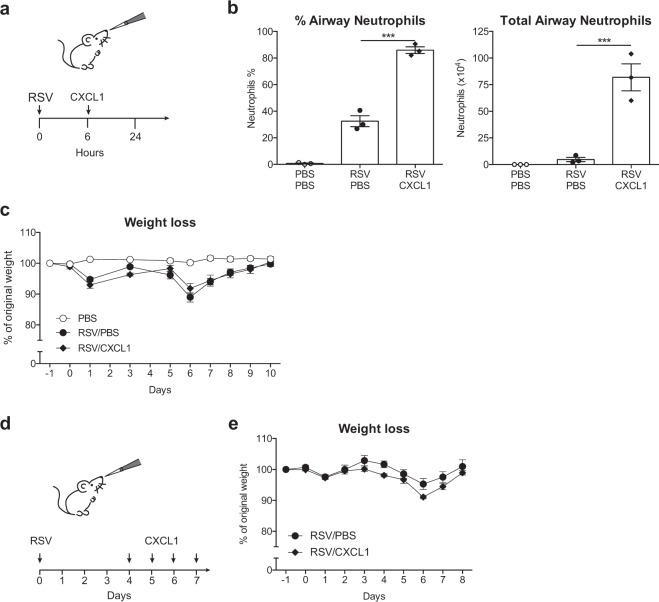
Accumulation of lung neutrophils during RSV infection does not affect weight loss. C57BL/6 or wt mice were mock (PBS) or RSV infected. (**a**) Lung neutrophils were increased by treatment with 10 µg CXCL1 i.n. 6 h p.i. (**b**) Frequency and total number of airway neutrophils 18 h p.i. (Supp. Figure 1 for full gating strategy). (**c**) Weight loss as percentage of original weight. (**d**) Lung neutrophils were increased by treatment with 5 µg CXCL1 i.n. day 4, 5, 6 and 7 p.i. (**e**) Weight loss as percentage of original weight. Data from (**b)** are presented as mean ± SEM of 3 individual mice per group from one experiment. Each symbol represents an individual mouse. Data from (**c)** are presented as mean ± SEM of 4 (PBS and RSV/CXCL1) and 7 (RSV/PBS) individual mice from one experiment. Data from (**e)** are presented as mean ± SEM of 4 (RSV/CXCL1) or 5 (RSV/PBS) individual mice from one experiment. For (**b)**, statistical significance of differences was determined by one-way ANOVA with Tukey’s post hoc test. For (**c**,**e**) statistical significance of differences was determined by two-way ANOVA with Bonferroni’s post hoc test. Only the statistical significances between RSV infected groups are shown. ***P ≤ 0.001.

We considered the possibility that the mouse model of RSV infection does not accurately mimic the massive influx of neutrophils observed clinically in infants with RSV-induced bronchiolitis^11^. Therefore, to enhance early neutrophil recruitment in our model, mice were treated i.n. with 10 μg recombinant CXCL1 6 h p.i. (Fig. 5a), which resulted in neutrophils constituting 90% of the cells in the airways (Fig. 5b), comparable to that observed in infants hospitalized with RSV induced bronchiolitis^11^. Notably, we found that neutrophil enhancement by treatment with rCXCL1 6 h p.i. did not influence disease severity as measured by weight loss during the infection (Fig. 5c). Next we wanted to test if the kinetics of lung neutrophil influx influences the severity of disease. In children with RSV induced bronchiolitis airway neutrophils are prominent during the peak of disease^24^. However, in mice, the primary recruitment of neutrophils occurs before weight loss develops^12,33^. To better model influx of neutrophils during the peak of RSV disease, mice were treated with 5 μg rCXCL1 i.n. on days 4, 5, 6 and 7 p.i. (Fig. 5d). However, again, exacerbating the influx of neutrophils even at the peak of disease did not drive increased weight loss (Fig. 5e). Thus, neutrophils infiltrating the lungs during RSV infection are not contributing to the severity of disease.

### Neutrophils are not necessary for the formation of T cell memory responses

**Figure 6:**
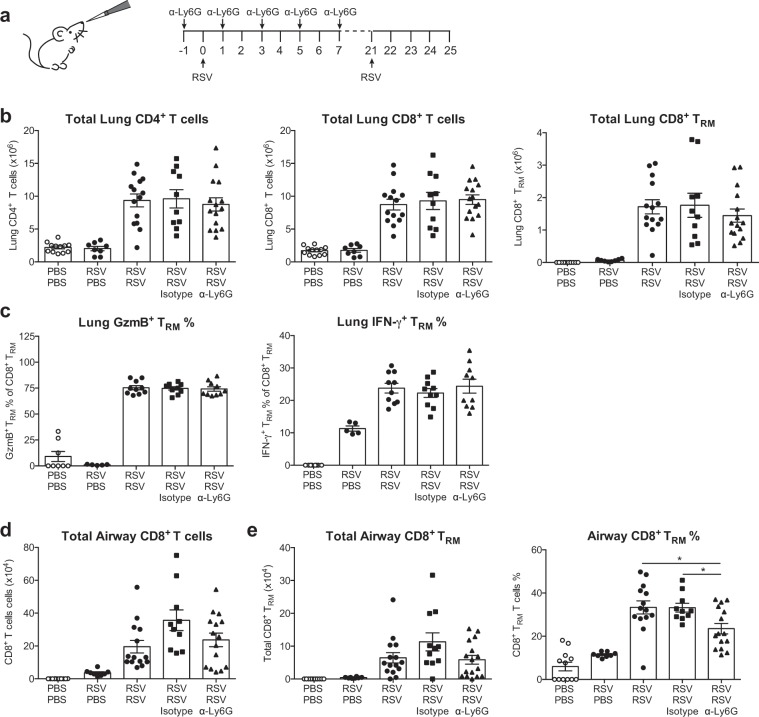
Lung neutrophils during primary RSV infection do not affect memory T cell responses upon re-challenge. (**a**) C57BL/6 or wt mice were mock (PBS) or RSV infected. To deplete neutrophils, 150 µg i.p. α-Ly6G or isotype control antibody was administered on days −1, 1, 3, 5 and 7. Mice were re-challenged with RSV on day 21. (**b**) Total numbers of lung CD4^+^ (CD19^−^, CD3^+^), CD8^+^ (CD19^−^, CD3^+^) and CD8^+^ T_RM_ (CD19^−^, CD3^+^, CD8^+^, CD62L^−^, CD44^+^, CD69^+^, CD103^+^; Supp. Figure 1 for full gating strategy). (**c**) Frequency of GzmB^+^ and IFN-γ^+^ producing CD8^+^ T_RM_ cells. (**d**) Total number of airway CD8^+^ (CD19^−^, CD3^+^) T cells, and **e**. total number and frequency of airway CD8^+^ T_RM_ (CD19^−^, CD3^+^, CD8^+^, CD62L^−^, CD44^+^, CD69^+^, CD103^+^). Each symbol represents an individual mouse. Data from (**b,d,e**) are presented as mean ± SEM of 8–15 individual mice pooled from two or three independent experiments. Data from (**c**) are presented as mean ± SEM of 5–10 individual mice from one or pooled from two independent experiments. Statistical significance of differences was determined by one-way ANOVA with Tukey’s post hoc test. Only the statistical significances between RSV re-infected groups are shown. *P ≤ 0.05.

Finally, we wanted to assess whether neutrophils influence the T cell memory response to RSV, as there is increasing evidence that neutrophils can influence adaptive and memory responses in other infectious disease scenarios^32,42^. Humans are unable to generate lasting, effective antibody responses to RSV and as such, memory T cell responses are especially important to protect against RSV re-infection^10,43,44^. Resident memory T cells (T_RM_) proliferate rapidly in the local tissue in response to re-challenge and can promote viral clearance^45^. To investigate whether neutrophils influence the generation of lung T_RM_ cells, mice were treated with α-Ly6G to abrogate neutrophil recruitment throughout the primary RSV infection (Fig. 6a). Mice were then re-challenged with RSV i.n. at 21 days p.i. and memory T cell responses were evaluated 4 days post re-challenge (Fig. 6a). Mice treated with α-Ly6G during the primary RSV infection had comparable recruitment of CD4^+^, CD8^+^ and CD8^+^ T_RM_ cells to the lungs as compared to both untreated, infected mice and to isotype control treated, infected mice (Fig. 6b). The frequencies of these cell populations were also unchanged between neutrophil depleted and isotype control treated, RSV infected groups (Supp. Figure 5). To assess T cell functionality in RSV infected mice, lung cells were stimulated with the RSV M peptide and cytokine production was assessed by intracellular flow cytometry (Fig. 6c). There was no difference in the ability of CD8^+^ T_RM_ cells to produce IFN-γ or GzmB following stimulation, suggesting that neutrophil depletion does not affect the functionality of this memory T cell subset (Fig. 6c). In the airways, there was no significant difference in the total number of CD8^+^ T cells or CD8^+^ T_RM_ cells in neutrophil depleted mice as compared to both untreated and isotype control treated RSV infected mice (Fig. 6d,e). However, the frequency of airway CD8^+^ T_RM_ cells was significantly reduced in neutrophil-depleted mice as compared to untreated and isotype control treated RSV infected mice (Fig. 6e). Thus, these data suggest that neutrophils are not majorly influencing the T cell responses during RSV infection.

## Discussion

Neutrophils have long been hypothesized to contribute to disease severity during severe RSV infection in infants^11,24^. Using a mouse model, we found that neutrophils do not act as major contributors to the pro-inflammatory environment or drive disease severity, despite their abundance in the lung following RSV infection. Furthermore, neutrophils do not appear to be an essential component of the host defense response as they do not restrict viral replication in the lungs. Overall, these data suggest that neutrophils are not exerting a beneficially anti-viral role during RSV infection.

We found that neutrophils do not impair the establishment of an anti-viral, inflammatory immune environment, which is dominated by the production of type I IFNs^12,40,46,47^. The induction of type I IFNs by alveolar macrophages (AMs) in response to RSV initiates the upregulation of hundreds of interferon stimulated genes (ISGs) encoding anti-viral mediators. This includes CCL2 which recruits anti-viral monocytes to the lung^12^. Neutrophil depletion did not impair the recruitment of monocytes early during RSV infection; in fact, neutrophil depletion increased the total number of monocytes in the lung during RSV. This has also been observed in a house dust mite model^48^. Furthermore, neutrophil depletion did not significantly inhibit the levels of IL-6, TNF-α or IL-1β as detected in the airways (in BAL fluid) confirming that neutrophils are not a major source of these mediators in the lung during RSV infection. AMs are a known source of these cytokines^49^, so these data also suggest that AM function during RSV infection does not depend on cross-talk with neutrophils. Recently, it was found that the release of mature IL-1β from AMs during influenza infection requires a neutrophil-derived peptide^22^. Together these studies highlight how the role of neutrophils varies between infections with different respiratory viruses.

TNF-α is an important cytokine during RSV infection, both for mediating RSV-induced illness and for activating cells such as neutrophils^35,50,51^. During RSV infection, inhibiting the binding of TNF to the TNFR using short peptides or antibody depletion of TNF decreased inflammation and illness^50–53^. The role of TNF in RSV control is not as clear as viral load was shown to decrease after blocking TNF using short TNF peptides^50^, but to increase when using antibody-depletion of TNF^51,53^. We did not find a change in TNF-α levels in the airways of neutrophil depleted mice. In disagreement with our data, a previous study showed a 2-fold decrease of TNF-α in lung homogenate 24 h post RSV infection after α-Ly6G antibody depletion of neutrophils in BALB/c mice using a different strain of RSV (2–20)^54^. The disparity between the two studies could suggest that the neutrophil response during RSV infection may differ depending on the mouse or viral strain used and the type of lung sample used for protein detection.

Most RSV studies using antibody-mediated neutrophil depletion do not include untreated, infected controls (e.g. references^22,23,54^). Notably, we found that the isotype control antibody seemed to have some off-target effects as it affected the cellular composition of the blood. We also found that RSV-infected, isotype control treated mice produced less IFN-α and IL-6 than the RSV only group. This should be taken into consideration when α-Ly6G is used to deplete neutrophils in other viral lung infections as well as during fungal and bacterial infections and during sterile inflammation. It is known that neutrophils regulate their own recruitment into tissues via a positive feedback loop by secreting MMP-9, a proteolytic enzyme which can degrade the chemoattractant CXCL1^55^. This was reflected in our data by our finding that neutrophil depletion resulted in increased production of CXCL1 in the airways of RSV infected mice.

Neutrophil depletion also demonstrated that neutrophils do not restrict viral replication during RSV infection of C57BL/6 mice *in vivo*. These findings are in agreement with studies showing no change in RSV or PVM load after neutrophil depletion, respectively^23,54^. As neutrophil defense mechanisms are largely extracellular or involve phagocytosis, it is not immediately clear how neutrophils would directly be anti-viral against RSV, especially as the virus is able to spread through the formation of syncytia^56^. It has been suggested that RSV can replicate in neutrophils^57^. However, our data suggest that RSV does not need neutrophils for its replication and that neutrophils are not a major cell target for viral production.

In our study, neutrophil depletion neither ameliorated nor potentiated the timing or severity of weight loss during RSV infection in either C57BL/6 or BALB/c mice. Weight loss is considered a readout of disease severity in mice and it is largely driven by CD8^+^ T cells^37^. Neutrophil depletion did not affect the recruitment of CD4^+^ or CD8^+^ T cell to the lung, nor was the formation of antigen-specific T cells in the lung altered. In light of this, it is perhaps not surprising that neutrophil depletion did not reduce weight loss in RSV infected mice. During influenza infection, neutrophils have been shown to guide the recruitment of T cells into the lung by leaving trails of the chemokine CXCL12^32^ and neutrophil depletion reduces the CD8^+^ T cell numbers in the lung^21^. Our data suggest that this mechanism is not conserved between respiratory viral infections.

The infiltration of airway neutrophils was less extreme in our *in vivo* model of RSV disease than observed clinically in infants with RSV-induced LRTIs^11^. Furthermore, infants present with masses of neutrophils during the peak of symptomatic disease while neutrophils are largely absent during the peak of disease severity in mice. Compensating for this by enhancing neutrophil numbers (using rCXCL1) in mice to frequencies comparable to those observed during severe disease in infants did not change the timing or severity of RSV-induced weight loss. Although surprising, these findings challenge the notion that neutrophils are, by default, destructive and demonstrate that even in an extremely delicate tissue such as the lung, the presence of large numbers of airway neutrophils can be tolerated without causing disease. This should also be considered in the context of fungal and bacterial infections which induce infiltration of neutrophils into the lungs. Increasingly, there is evidence to suggest that neutrophils interact with the adaptive immune system and may influence memory responses^58^. Neutrophil depletion throughout the primary infection did not have a major effect on the generation of memory T cell or T_RM_ cell responses detected during RSV re-challenge. Although we observed a slight decrease in the T_RM_ cell frequency in the airways of neutrophil depleted mice after RSV re-challenge, there was no change in the total number of airway T_RM_ cells.

While neutrophils are an important line of defense during many inflammatory conditions, their recruitment and activation must be tightly regulated to prevent bystander damage on host tissues^17^. In a previous study we have shown that the inflammatory environment in the lung induced during RSV infection drives neutrophil activation^33^. However, we could not detect any major differences in weight loss after neutrophil depletion during RSV infection. In addition, we could not detect any differences in mucus production after neutrophil depletion, although, in a study using a different RSV strain (2–20), neutrophil depletion decreased mucin production at day 8 p.i^54^. It remains possible that neutrophils contribute to lung pathology during RSV infection that does not translate into weight loss. Indeed, neutrophils contribute to disease severity during infection with numerous influenza virus strains^20,59^. However, not all studies on influenza virus infection after neutrophil depletion are in agreement on whether neutrophils are beneficial (anti-viral) or detrimental (causing lung damage)^20,21,60–62^. These discrepancies may be due to the viral strain or dose used^60^. It is therefore possible that lung pathology and mucin production are influenced by neutrophils in slightly different ways dependent on which mouse and infection models and type of viral strains used.

As an approach, neutrophil depletion is relatively crude and has the limitation that it removes all the effects of neutrophils, some of which may be beneficial and some detrimental to disease. This might mean that subtle anti-viral effects exerted by neutrophils are not observed in this system. Our data suggest that neutrophil infiltration is not essential for control of viral replication by the host during RSV nor that neutrophil recruitment drives weight loss during RSV infection of mice. These data exclude a beneficial anti-viral role for neutrophils in RSV infection, yet we remain uncertain as to whether neutrophils can cause lung pathology or impact the resolution phase. RSV infection induces neutrophil activation and degranulation, causing the secretion of proteolytic enzymes into the lung^33^. Furthermore, it has been demonstrated that RSV likely induces NETosis^63,64^. Both these functions of neutrophils can cause tissue damage^65^ and the extent to which these functions damage the lung during RSV infection *in vivo* so as to affect recovery from infection is yet to be fully elucidated.

Our findings suggest that neutrophils are not required for the induction of an anti-viral inflammatory environment and do not appear important for the control of viral replication in the lungs during RSV infection of C57BL/6 mice. Most surprisingly, we did not find that removal or exacerbation of the number of airway neutrophils had consequences on either the timing or severity of RSV induced disease measured by weight loss. Overall, our data suggest that the potentially pathogenic bystander effects of neutrophils can be controlled and tolerated in the lung during RSV infection.

## Materials and Methods

### Mice

C57BL/6 and BALB/c mice were purchased from Charles River. *Ifna6gfp*^+/−^ mice on a SJL/C57BL/6 background were bred in-house (obtained from S. Akira, Japan^66^). As the GFP signal has not been quantified in this work the mice are noted as wt throughout. All mice were bred and housed in specific pathogen-free conditions and were gender and age-matched (7–12 weeks). All animal experiments were reviewed and approved by the Animal Welfare and Ethical Review Board (AWERB) within Imperial College London and approved by the UK Home Office in accordance with the Animals (Scientific Procedures) Act 1986 and the ARRIVE guidelines.

### Virus and infections

Plaque-purified human RSV (originally A2 strain from ATCC, US) was grown in HEp2 cells^67^. Mice were lightly anaesthetized before administration intranasally (i.n.) of 100 µl containing 7.5–10 × 10^6^ FFU RSV or PBS control. In some instances, this was followed by i.n. instillation of 5 µg or 10 µg recombinant CXCL1 (Biolegend). Mice were sacrificed post-infection by a fatal dose of pentobarbital injected intraperitonially (i.p.).

### Antibody mediated neutrophil depletion

Two regimes were used to deplete neutrophils in mice, as indicated in figure legends. Mice were either given 200 µg i.n. and 500 µg i.p. α-Ly6G MAb or isotype rat Ig2A MAb (Bio X Cell) one day pre-infection or mice were given 150 µg i.p. one day pre-infection and then every other day throughout the infection as indicated in the figure legends^20,22,32^. The isotype control was always given the same route, dose and frequency as the α-Ly6G Mab.

### Airway cell processing

To recover the airway cells and immune mediators, BAL was performed^68^. One ml PBS supplemented with 0.5 mM EDTA was used to flush the lungs three times. The BAL was centrifuged, and supernatant stored at −80 °C. The cells were incubated 2 min in ACK buffer (0.15 M NH_4_Cl, 1.0 mM KHCO_3_, 0.1 mM Na_2_EDTA) to lyse red blood cells (RBCs) and the cell number was determined by Trypan Blue (Thermo Fisher Scientific) exclusion of dead cells. Cells obtained from the BAL were termed airway cells throughout. Airway cells were either stained for flow cytometry or the cellular composition was determined by spinning 1–2 × 10^5^ cells onto a microscope slide (Thermo Scientific) at 450 rpm for 5 min using Cytospin 4 Cytocentrifuge (Thermo Fisher Scientific). Slides were H&E stained using Reastain Quick-Diff kit (Gentaur), according to the manufacturer’s instructions. Neutrophils were imaged using a light microscope and determined by nuclear morphology.

### Isolation of lung cells and peripheral mononuclear blood cells

For RNA extractions, lung lobes were snap-frozen in liquid nitrogen. For flow cytometry, a single cell lung preparation was obtained as previously described^33^. At least 75 µl blood was collected in 1 ml PBS supplemented with 5 mM EDTA. RBCs were removed by lysis with ACK. Cells were re-suspended in FACS buffer (PBS supplemented with 1% BSA, 0.05 mM EDTA) before flow cytometry staining.

### Flow cytometry

For flow cytometry staining, 2.5 × 10^6^ lung, airway or blood cells were incubated for 20 min at 4 °C with a purified rat IgG2b α–mouse CD16/CD32 receptor antibody (BD) to block Fc binding in FACS buffer. For surface staining, cells were stained with fluorochrome-conjugated antibodies against CD45 (30-F11, BV605/APC eFluor 780), CD11b (M1/70, AF700), CD64 (X54-5/7.1, FITC), Ly6G (1A8, BV570/FITC), CD3 (17A2, AF700), Ly6C (HK1.4, eFluor450), CD19 (6D5, FITC), CD69 (H1.2F3, BUV737), CD62L (MEL-14, BV421), CD11c (HL3, PE-CF594), CD4 (GK1.5, PE), CD8 (53–6.7, eFluor 780), CD103 (2E7, PerCP-Cy5.5), GzmB (GB11, PE-CF594), IFN-γ (XMG1.2, BV711) for 25 min at 4 °C. Cells were washed with PBS and stained with fixable live-dead Aqua dye (Invitrogen) for 30 min. Cells were fixed by incubation with 100 µl 1% paraformaldehyde or Cytofix™ Fixation Buffer (BD) for 20 min at 4 °C and stored in FACS buffer. For RSV M Tetramer staining, Alexa Fluor 647-conjugated M_187–195_ tetramers (H-2Db/NAITNAKII) were obtained from the NIH Tetramer Core Facility (Emory University, Atlanta, GA, USA). Tetramer staining was performed following Fc-block and prior to surface staining for 30 min in PBS containing 1% BSA and 5 mM EDTA. For intracellular staining the cells were re-stimulated with 5 μg/ml RSV M_187–195_ peptide for 1 h at 37 °C. After addition of Golgi Plug (BD Biosciences) the samples were incubated for another 3 h, stained for surface marker expression as described above and fixed in fixation buffer (Biolegend). Cells were stained with fluorochrome-conjugated antibodies against granzyme B (GB11, PE-CF594) and IFN-γ (XMG1.2, BV711) in the presence of purified rat IgG2b anti-mouse CD16/CD32 receptor antibody in permeabilization buffer (Biolegend) for 1 h. Analysis was performed on a BD LSR Fortessa. Acquisition was set to 250,000 single, live, CD45^+^ cells. All antibodies were purchased from BD, BioLegend, or eBioscience. Data were analyzed with FlowJo software (Tree Star). Total cell populations were quantified as the whole lung count x (%population of CD45^+^ cells) x (%CD45^+^ cell of live cells) x (proportion of lung tissue sampled).

### RNA extraction, quantitative RT-PCR and viral load determination

Lungs were homogenized using a TissueLyser LT (Qiagen) and total RNA was extracted from the lung tissue supernatant using RNeasy Mini kit (Qiagen). RNA concentration was determined by NanoDrop (Thermo Scientific). 2 µg of RNA was converted to cDNA using High Capacity RNA-to-cDNA kit (Applied Biosystems). RT-qPCR was performed with Quantitect Probe PCR Master Mix (Qiagen). RSV L gene mRNA was quantified as previously described^12^. For exact quantification, copy numbers were calculated from a plasmid DNA standard curve and normalized to *Gapdh* (encoding glyceraldehyde-3-phosphate dehydrogenase). For mRNA analysis of *Gapdh, Cxcl1, Cxcl2, Cxcl12, Il1a, Il1b, Il33, Areg*, *Ptgs2*, *Muc5ac* gene specific primers and probes were used (all Applied Biosystems). RT-qPCR was performed using the 7500 Fast Real-Time PCR System (Applied Biosystems). To quantify relative mRNA expression the mean ΔCT was calculated for each target gene relative to *Gapdh* and expressed as 2^−ΔCT^. Analysis was performed using 7500 Fast System SDS Software (Applied Biosystems).

Live virus was quantified in the lungs by immunoplaque assay as previously described^12,36^.

### Immune mediator detection

ELISA was used to measure the concentration of IL-1β (eBioscience), TNF-α, CXCL1 (R&D systems DuoSet), IL-6^40^ and IFN-α^40^. Absorbance was determined at 450 nm, on SpectraMax Plus (Molecular Devices) or FLUOstar Omega (BMG Labtech) plate readers and analyzed using SoftMax (Molecular Devices) or Mars (BMG Labtech) software.

### Statistical analysis

Statistical analysis was performed using Prism (GraphPad software) version 6. Data are presented as the mean ± SEM. As indicated in the figure legends, Student *t* test, one-way ANOVA followed by Tukey’s post hoc or a two-way ANOVA followed by Bonferroni’s post hoc test was used to determine statistical significance between groups. P values <0.05 were considered statistically significant for all tests. *P < 0.05; **P < 0.01; ***P < 0.001.

## Supplementary information

Supplementary info.
