# Supplementary material for: Neutrophils do not impact viral load or the peak of disease severity during RSV infection
Source: Sci Rep. 2020 Jan 24;10:1110. doi: 10.1038/s41598-020-57969-w (PMC6981203; doi:10.1038/s41598-020-57969-w)
Supplement: Supplementary file 1 — Supplementary info. [file 41598_2020_57969_MOESM1_ESM.pdf]

Supplementary information for:

Neutrophils do not impact viral load or the peak of disease severity during RSV infection

Freja Kirsebom, Christina Michalaki, Marina Agueda-Oyarzabal and Cecilia Johansson

Neutrophils do not impact viral load or the peak of disease severity during RSV infection

Kirsebom et al

Supplementary Figures

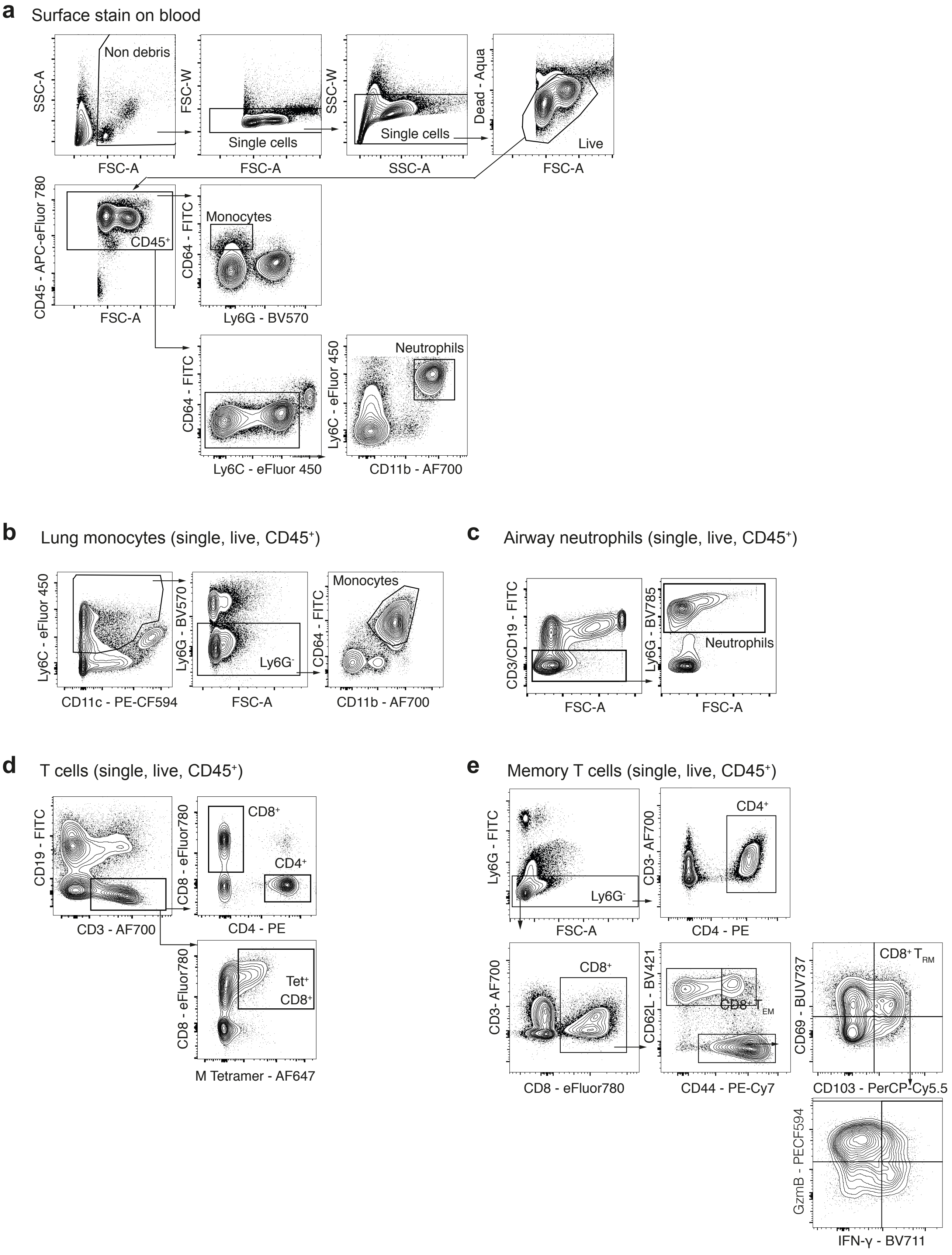

Supplementary Figure 1. Gating strategies for identifying airway and lung immune cells. Mice were intranasally infected with RSV. **a.** Blood cells were stained for the indicated cell surface molecules. After excluding debris and gating on single, live, $CD45^+$ cells, monocytes and neutrophils were identified. Lung immune cells were obtained by collagenase digestion and lung and airway cells were stained for the indicated cell surface molecules. After excluding debris and gating on single, live, $CD45^+$ cells, the depicted gates were used to identify **b.** monocytes and **c.** neutrophils **d.** $CD8^+$, $CD4^+$ and $CD8^+$ Tet^+ T cells and **e.** memory T cells and GzmB and IFN-γ producing cells.

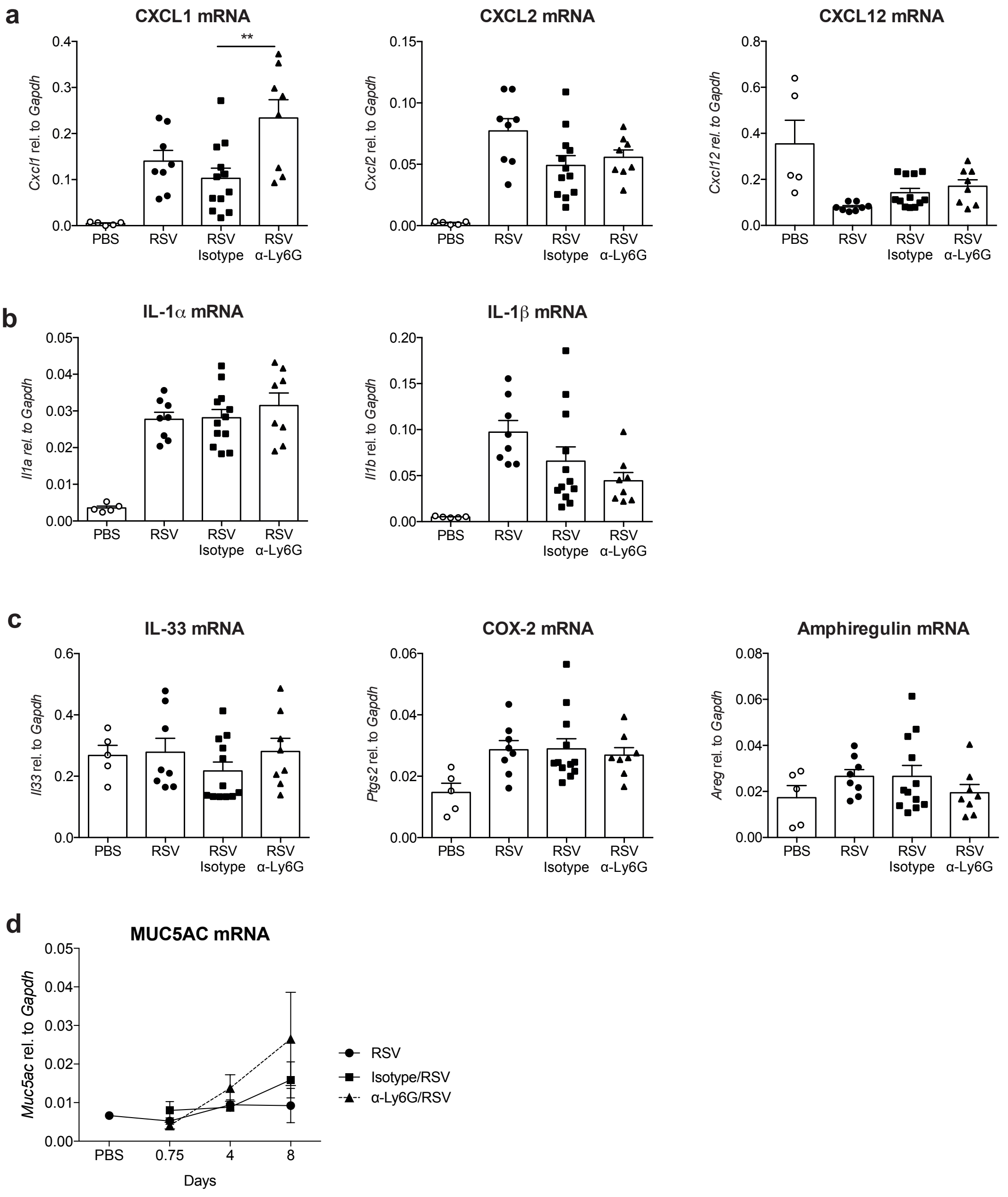

Supplementary Figure 2. Neutrophils do not influence the early (18h post infection) gene expression of pro-inflammatory mediators or *Muc5ac* in the lungs post RSV infection. a-c. Wt mice were mock (PBS) or RSV infected for 18h. To deplete neutrophils, RSV infected mice were given 200 µg i.n. and 500 µg i.p. α-Ly6G or isotype control antibody day -1. *Cxcl1, Cxcl2*, *Cxcl12, Il1a, Il1b, Il33, Ptgs2* and *Areg* mRNA were quantified in the lung by RT-qPCR. **d.** Wt mice were mock (PBS) or RSV infected. To deplete neutrophils, 150 µg i.p. α-Ly6G or isotype control antibody was administered on day -1 and on every second day throughout the infection. PBS mice are pooled from each time point. *Muc5ac*

was quantified in the lung by RT-qPCR. The relative expression ($2^{-\Delta CT}$) is expressed normalized to *Gapdh.* In **a-c**, data are presented as the mean±SEM from 5 (PBS) or 8-12 (RSV) individual mice pooled from two or three independent experiments. Each symbol represents an individual mouse. In **d**, data are presented as the mean±SEM from 20 (PBS) or 8-12 (RSV) pooled from two to three independent experiments (18h and 4 days) or 4-5 (RSV) from one experiment (day 8). Statistical significance of differences was determined by one-way ANOVA with Tukey's post hoc test. Only the statistical significances between RSV infected groups are shown. ** P ≤ 0.001.

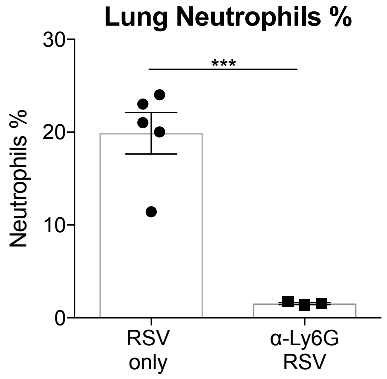

Supplementary Figure 3. Depletion of lung neutrophils. To confirm depletion of lung neutrophils using the lower dose of α-Ly6G antibody, wt mice were given 100 µg i.p. α-Ly6G antibody on day before infection. Mice were infected with RSV for 18h and lung neutrophils were quantified by flow cytometry (not gated on Ly6G). The data are presented as the mean±SEM from 3-5 mice. Each symbol represents an individual mouse. Statistical significance of differences was determined by an unpaired Student's *t* test. *** P ≤ 0.01.

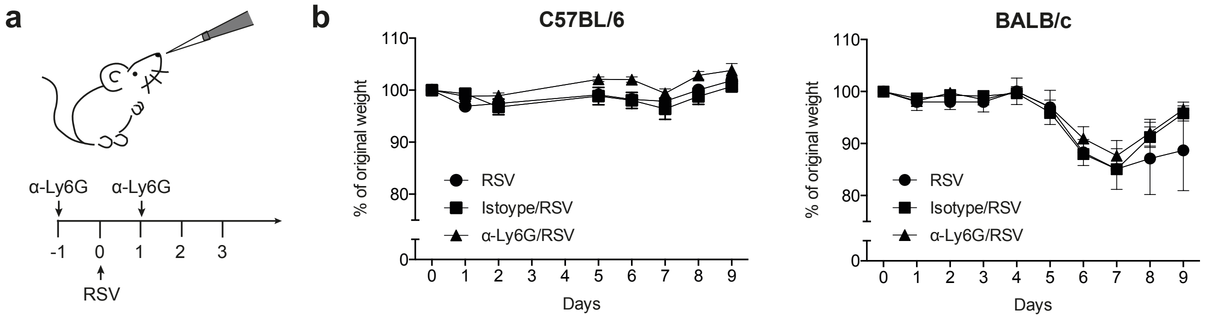

Supplementary Figure 4. Neutrophils do not influence the weight loss of either C57BL/6 mice or BALB/c mice during RSV infection. a. Wt and BALB/c mice were mock (PBS) or RSV infected. To deplete neutrophils, 500 μg i.p. and 200 μg i.n. α-Ly6G or isotype control antibody was administered on day -1 and day 1 p.i.. **b.** Weight loss as percentage of original weight during RSV infection in C57BL/6 and BALB/c mice. Data are presented as the mean±SEM from 2-4 (C57BL/6) or 3-5 (BALB/c) individual mice from one experiment. Statistical significance of differences was determined by two-way ANOVA with Bonferroni's post hoc test.

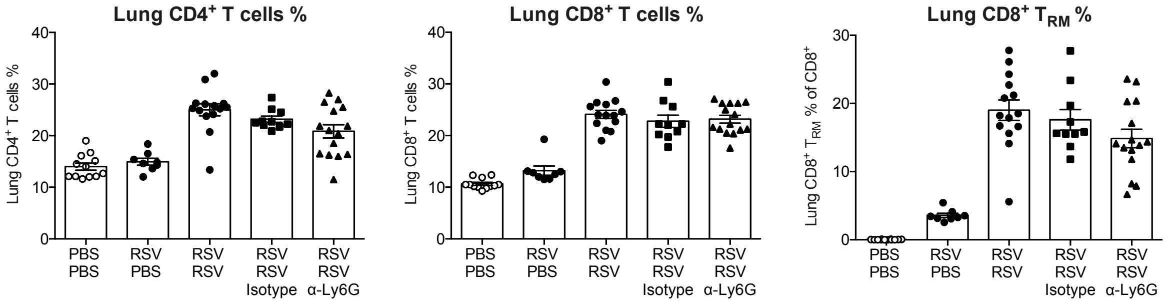

Supplementary Figure 5. Infiltration of neutrophils during primary infection does not influence the frequencies of T cells in the lung after RSV re-challenge. C57BL/6 or wt mice were mock (PBS) or RSV infected. To deplete neutrophils, 150 µg i.p. α-Ly6G or isotype control antibody was administered on days -1, 1, 3, 5 and 7. Mice were re-challenged with RSV on day 21. Frequencies of lung $CD4^+$ ($CD19^-$, $CD3^+$), $CD8^+$ ($CD19^-$, $CD3^+$) and $CD8^+$ T_{RM} ($CD19^-$, $CD3^+$, $CD8^+$, $CD62L^-$, $CD44^+$, $CD69^+$, $CD103^+$) (Supp. Fig. 1 for full gating strategy). Data are presented as mean±SEM of 8-15 individual mice pooled from two or three independent experiments. Each symbol represents an individual mouse. Statistical significance of differences was determined by one-way ANOVA with Tukey's post hoc test.
